# Insight Into Polysaccharides From *Panax ginseng* C. A. Meyer in Improving Intestinal Inflammation: Modulating Intestinal Microbiota and Autophagy

**DOI:** 10.3389/fimmu.2021.683911

**Published:** 2021-07-20

**Authors:** Dandan Wang, Shuai Shao, Yanqiu Zhang, Daqing Zhao, Mingxing Wang

**Affiliations:** ^1^ College of Chinese Medicine, Changchun University of Chinese Medicine, Changchun, China; ^2^ Jilin Provincial Key Laboratory of BioMacromolecules of Chinese Medicine, Jilin Ginseng Academy, Changchun University of Chinese Medicine, Changchun, China; ^3^ College of Pharmacy, Changchun University of Chinese Medicine, Changchun, China; ^4^ Jilin Ginseng Academy, Changchun University of Chinese Medicine, Changchun, China

**Keywords:** *Panax ginseng* C. A. Meyer, polysaccharide, intestinal inflammation, gut microbiota, autophagy

## Abstract

Polysaccharides from *Panax ginseng* C. A. Meyer (P. ginseng) are the main active component of P. ginseng and exhibit significant intestinal anti-inflammatory activity. However, the therapeutic mechanism of the ginseng polysaccharide is unclear, and this hinders the application for medicine or functional food. In this study, a polysaccharide was isolated from P. ginseng (GP). The primary structure and morphology of the GP were studied by HPLC, FT-IR spectroscopy, and scanning electron microscopy (SEM). Further, its intestinal anti-inflammatory activity and its mechanism of function were evaluated in experimental systems using DSS-induced rats, fecal microbiota transplantation (FMT), and LPS-stimulated HT-29 cells. Results showed that GP modulated the structure of gut microbiota and restored mTOR-dependent autophagic dysfunction. Consequently, active autophagy suppressed inflammation through the inhibition of NF-κB, oxidative stress, and the release of cytokines. Therefore, our research provides a rationale for future investigations into the relationship between microbiota and autophagy and revealed the therapeutic potential of GP for inflammatory bowel disease.

## Introduction

Inflammatory bowel disease (IBD) consists of a group of disorders including Crohn’s disease (CD) and ulcerative colitis (UC) and is a kind of recurrent, refractory gastrointestinal disease (1). At present, the incidence of IBD increases yearly. Inflammatory infiltration, redox imbalance, and gut microbiota dysbiosis are involved in the initiation, development, and exacerbation of intestinal inflammatory diseases. Intestinal epithelium plays an important role in maintaining gut homeostasis and is the main defense against pathogen invasion (2). It has been reported that the regulation of gut microbiota can alleviate the inflammatory response induced by dextran sodium sulphate (DSS) in mice. Lipopolysaccharide (LPS) is the main component of the cell walls of many Gram-negative bacteria. The inseparable relationship between microbiota dysbiosis, LPS content, and abnormal immune response has been reported previously (3, 4). Toll-like receptor 4 (TLR4), which is abundantly expressed in intestinal epithelial cells, is a gene coding receptor for bacterial LPS (5, 6). An abnormal microbiome, especially with microbes that produce LPS, triggers intestinal inflammation, and TLR4 might be the initial point of microbial interaction. Activated TLR4 recruits the downstream molecule MyD88 to trigger the phosphorylation of MAPKs (7), and it is indispensable in orchestrating the secretion of inflammatory cytokines and oxidative stress response throughout the initiation, development, and exacerbation of IBD (8–10).

Autophagy, a highly conserved process that evolved in eukaryotes, is involved in maintaining organism homeostasis *via* lysosome-mediated self-digestion and recycling of organelles and proteins (11, 12). Cells trigger autophagy under various stress, such as exposure to toxic environments, starvation, and ischemiareperfusion (13). It has been reported that autophagy dysfunction increased susceptibility to inflammatory intestinal diseases (14–17). Repairing hampered autophagy normalized redox imbalance, increased the clearance of intracellular bacteria, and alleviated inflammation in intestinal mucosa (18); thus, it has become a new target of clinical drug development for IBD. Mounting evidence suggests the inseparable association between autophagy impairment and inflammation injury (19–21). mTOR, a highly conserved serine/threonine protein kinase, negatively regulates autophagy upstream, and it has demonstrated great autophagy activating and inflammation attenuating efficacy in silencing mTOR (22). Interestingly, TLR4-MyD88-MAPK is one of the important pathways in mTOR regulation, and recent research clearly revealed the relationship between them (19). Accumulating evidence indicates that autophagy and inflammation are linked by reciprocal regulation through the microflora-TLR4-mTOR axis. Mechanistically, microbiota dysbiosis activates the TLR4-MyD88-MAPK pathway, which is followed by the phosphorylation of mTOR to inhibit autophagy, thereby aggravating inflammatory injury and oxidative stress (19). Although the roles of microflora in modulating inflammation and autophagy have had increased attention in regard to IBD, the development of related drugs is still in its infancy.

*Panax ginseng* C. A. Meyer (P. ginseng) has been widely used as an herb and functional food in the world (23). It has been reported that the active components of *Panax ginseng* have anti-inflammatory and immunomodulatory effects on IBD (24). The polysaccharide of P. ginseng has obvious beneficial effects, including gut microbiota regulation, intestinal mucosal barrier protection, autophagy promotion, and alleviation of inflammation and oxidative stress (24). However, the exact target and mechanism of the P. ginseng polysaccharide in gut microbiota and autophagy modulation is not well understood. Fecal microbiota transplantation (FMT) is one of the most effective ways to regulate the gut microbiota and can potentially reveal the function of microbiota and establish the causal relationship between flora and disease (25). At present, the use of FMT with P. ginseng polysaccharide to treat diseases through intestinal flora is still unknown. Therefore, the present study aims to purify crude polysaccharides from P. ginseng and to evaluate its gut microbiota and intestinal anti-inflammatory activity in dextran sulfate sodium (DSS)-induced rats with FMT. The mechanism of reciprocal regulation between inflammation and autophagy was estimated by LPS-induced inflammatory intestinal mucosal cells (HT-29 cells). Additionally, the changes in cytokines, reactive oxygen species (ROS), and autophagic proteins were detected in order to investigate the protective mechanism of the microbiota-autophagy relationship. Our study provides a reliable theoretical basis for the application of ginseng polysaccharide as a functional food material in the treatment of intestinal diseases.

## Materials and Methods

### Reagents

P. ginseng was purchased from Wudu County (Gansu Province, China). The roots of P. ginseng were dried in the shade and ground into powder. Standard monosaccharides, including rhamnose, arabinose, xylose, mannose, glucose, galactose, glucuronic acid, and galacturonic acid were all obtained from the National Institute for the Control of Pharmaceutical and Biological Products (Beijing, China). All chemicals used were of analytical grade unless otherwise specified.

### Isolation and Purification of Polysaccharide

Briefly, the dry three-year roots of Radix ginseng (2,000 g) were smashed into crude powder (60-80 mesh) and extracted three times with water (20 L) by continuously stirring at 80°C for two hours each time. The combined aqueous extracts were filtered through a cotton cloth bag and centrifuged (1,500 g for 15 min) and were subsequently concentrated in a rotary evaporator at 60°C. Then, the ethanol solution was brought to a final concentration of 80%. The sediment was dissolved in water, continuously stirred at 4°C for 12 hours, and the precipitate was removed by centrifugation (1,500 g for 15 min). The aqueous extract was then filtered and concentrated, followed by the addition of Sevage reagent (butanol and chloroform at a 1:4 ratio) to deproteinate the sample (26). Then, the supernatant was concentrated to a proper volume under reduced pressure and lyophilized to get the ginseng polysaccharide (GP).

### Analysis of the Primary Structure and Morphological Properties of GP

#### Analysis of Chemical and Physical Properties

The total carbohydrate, uronic acid, and protein contents were quantified by the phenol–sulfuric acid method (27), m-hydroxydiphenyl (28), and Bradford method (29) using glucose, glucuronic acid, and bovine serum albumin as standards, respectively.

#### Monosaccharide Composition Analysis

The monosaccharide components were analyzed by converting the sugars into PMP derivatives (30), which were then detected by HPLC (Shimadzu 2010, Japan) and C18 column (Shimadzu, Japan) and controlled by a Uniport HP N-2000 data station.

#### FT-IR Analysis

The vibrations of molecules and polar bonds among different atoms were studied by FT-IR spectroscopy (iD1 Transmission Nicolet iS5, Thermo Fisher, USA), with recorded frequencies ranging from 4,000 to 500 cm^-1^ (31). FT-IR measurements were performed for each sample, which were mixed with dried KBr powder and then compressed into salt discs.

#### Molecular Weight Analysis

The molecular weight of the GP was determined by a gel-permeation chromatography (GPC) system (ELEOS System, Wyatt Technology, USA) equipped with a Waters 515 pump and DRI detector. The columns were a Shodex OHpak SB-806 in sequence with 803 columns, which were eluted with 0.02% NaNO_3_ at a flow rate of one mL/min. The temperatures of the column and RI detector were maintained at 40°C. Dextran standards (Mw = 180, 2,700, 9,750, 36,800, and 135,350 Da) were used to establish a standard curve. Empower GPC software (ASTRA5.3.4, Wyatt Technology, USA) was used for data processing (32).

#### Scanning Electron Microscopy (SEM) Analysis of Morphological Properties

The micro-structures and surface morphologies of GP were characterized by scanning electron microscopy (SEM). A proper amount of polysaccharide sample powder was glued to the conductive adhesive of the experimental bench. The floating sample was blown off with a rubber suction bulb and placed in a gold conductive layer of 10 nm thickness in a vacuum. The accelerating voltage of the electron gun was 20 KV, and the sample was observed by the SEM.

### Animals

The animal experiment protocol (No. 20200128006) was approved by the Changchun University of Chinese Medicine. SD rats weighing 200-250 g (half of which were male) were purchased from the School of Pharmacy, Jilin University (SCXK 2016-0001). All rats were given standard food and water for seven days prior to experimentation and were randomly divided into the blank group (control), intestinal inflammation group (DSS) (19, 22), low-dose GP treatment group (DSS + GP-L, 50mg/kg), middle-dose GP treatment group (DSS + GP-M, 100mg/kg), and high-dose GP treatment group (DSS + GP-H, 200mg/kg); each group consisted of 10 rats. Experimental procedures used for the animals and administration methods were based on previously reported experimental protocols (33). The rats fasted for 12 hours on the last day, and each rat was anesthetized and euthanized. The blood from each rat was collected from the common carotid artery. Ten grams of fresh feces from the rats in each group were collected and weighed in a sterilized beaker, and 50 ml of 37°C sterile normal saline was added, stirred, and filtered with double-layer sterile gauze. The filtrate was then centrifugated at 6,000 g/min for 15 min (centrifugation radius: 10 cm), and then the sediment was suspended in 100 ml of normal saline to obtain the fecal bacteria solution. FMT (5 ml/kg) was administered by gavage once a day for 14 days. The distal parts of the colon tissues were stored in a -80°C refrigerator for section or Western blot analysis. Feces were collected for further metabolite analysis. Blood was centrifuged, and the supernatant was stored at -20°C for later testing. The relative serum levels of IL-1β, IL-6, and TNF-α were determined using ELISA kits (Beyotime Institute of Biotechnology, Shanghai, China) according to the manufacturer’s instructions.

### Immunohistochemistry Analysis of Intestinal Tissues

Immunohistochemistry analysis was conducted to evaluate the expression of relative proteins. The paraffin-embedded tissue sections were deparaffinized and treated with hydrogen peroxide (3 m/v) for 15 min to remove endogenous peroxidase. Antigen retrieval was performed by blocking the samples in goat serum for 10 min at 22°C. The following antibodies were added and incubated for 12 hours at 4°C: anti-TLR4 antibody (Servicebio, GB11519, 1:500), anti-LC3B antibody (Abcam, ab86714, 1:500), and anti-NF-κB p65 antibody (Servicebio, GB13025-1, 1:500). Cy3 (Servicebio, GB21303) and FITC (Servicebio, GB22303) secondary antibodies (1:250) were used for visualization. Tissue sections were mounted and analyzed using an inverted fluorescence microscope (Nikon Eclipse TI-SR) and imaging system (Nikon DS-U3).

### The Measurement of Endotoxin

Colon contents endotoxins were extracted in with 0.05% Tween-20 in pyrogen-free water (34). Fecal endotoxins were determined using Limulus amaebocyte lysate (LAL) kit (ThermoFisher, USA) as described previously (35).

### Gut Microbiota Analysis

Gut microbiota was examined as before by 16S ribosomal RNA analysis on the Illumina MiSeq platform (Illumina, San Diego, USA) according to standard protocols (36). DNA was extracted from the colon using an Omega Biotek Mag-Bind Soil DNA Kit (Omega Bio-Tek, USA). Purified PCR products were prepared using Q5^®^ High-Fidelity DNA Polymerase (NEB, USA); products were then quantified, and each PCR sample was diluted five times to 20 ng/μL. The PCR amplification system consisted of the following: PCR mixed product sample (2 µL), 5× reaction buffer (5 μL), 5× GC buffer (5 μL), dNTP (2.5 mM 2 μL), forward primer (10 µM, 1 μL), reverse primer (10 µM, 1 μL), Q5 DNA Polymerase (0.25 μL), DNA template (2 μL), and 8.75 μL ddH_2_O. PCR amplification of the 16S rRNA genes of the V3–V4 region was performed using the forward primer 338F 5’-ACTCCTACGGGAGGCAGCA-3’ and the reverse primer 806R 5’-GGACTACHVGGGTWTCTAAT-3’. Sample-specific 7-bp barcodes were incorporated into the primers for multiplex sequencing. The PCR components contained 5 μl of Q5 reaction buffer (5×), 5 μl of Q5 High-Fidelity GC buffer (5×), 0.25 μl of Q5 High-Fidelity DNA Polymerase (5 U/μl), 2 μl (2.5 mM) of dNTPs, 1 μl (10 uM) of each forward and reverse primer, 2 μl of DNA template, and 8.75 μl of ddH_2_O. Thermal cycling consisted of initial denaturation at 98°C for 2 min, followed by 25 cycles of denaturation at 98°C for 15 s, annealing at 55°C for 30 s, and extension at 72°C for 30 s, with a final extension of 5 min at 72°C. The amplicon library was then used for paired-end sequencing (2 × 250 bp) on an Illumina MiSeq platform (Illumina, San Diego, USA) according to standard protocols.

### Cell Culture and Treatment

The human epithelial colorectal adenocarcinoma HT-29 cell line was obtained from the Procell Life Science and Technology Company and was maintained in DMEM with 10% FBS, 1% nonessential amino acids, and gentamicin (50 μg/mL) at 37°C and 5% CO_2_; the medium was replaced every two days. For parameter measurements, the culture medium was seeded onto 6-well plates at a density of 5 × 10^5^ cells/well (Corning Inc., Corning, NY, USA). The cells were incubated with 1 mM 3-MA or 10 μM rapamycin for 1 hour. To induce inflammatory damage, the cells were exposed to LPS (1 μg/ml) at 37°C for 6 hours. HT-29 cells were transfected with 10 pmol of siRNA for TLR4 using Lipofectamine RNAiMAX according to the manufacturer’s instructions. The cellular proteins were obtained for western blot analysis as previously described (37).

### Western Blotting

For Western blot analysis, the total proteins from the colon cells were collected in RIPA lysis buffer (Beyotime, Beijing, China). Samples (40 μg each) were separated onto 12% SDS-PAGE gels and transferred to a PVDF membrane (Millipore Corp., Billerica, MA, USA). Membranes were incubated overnight with related primary antibodies at a 1:1000 dilution.

### Statistical Analysis

Experiments were performed in triplicate, and results are presented as the mean ± standard deviation (SD). The Student’s t-test was utilized for statistical evaluation using the SPSS 16.0 statistical software package (SPSS Inc., USA), and a *P* < 0.05 was considered statistically significant.

## Results

### Characterization of Polysaccharide

#### Isolation and Determination of GP

**Figure 1:**
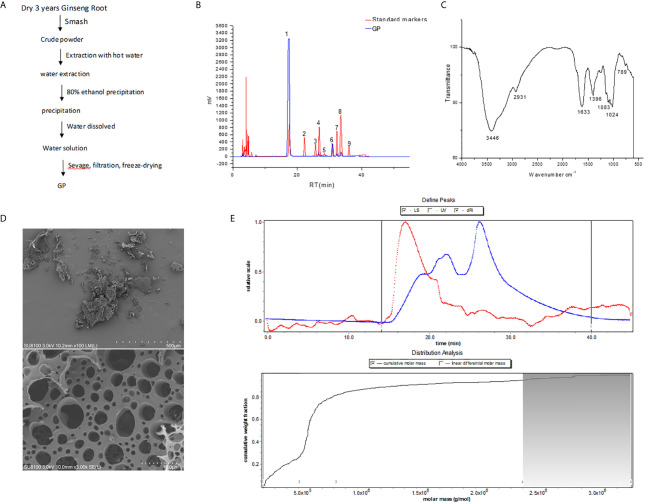
Description and analysis of GP **(A)** Summarized extraction procedure. **(B)** HPLC of monosaccharide PMP derivatization. 1. PMP. 2. Mannose. 3. Rhamnose. 4. Glucuronic acid. 5. Galacturonic acid. 6. Glucose. 7. Galactose. 8. Arabinose. 9. Fucose. **(C)** Infrared spectroscopy. **(D)** Morphology by SEM. **(E)** Molecular weight distribution.

GP were extracted by using the classical method of hot water extraction followed by ethanol precipitation. Then, Sevage reagent was used to deproteinate the aqueous extracts. The crude polysaccharide yield devoid of proteins was found to be 7.5% of the total dry weight. In addition, the polysaccharides were dissolved in water after alcohol precipitation, and the water solubility of the polysaccharides could be greatly increased. The extraction procedure is shown in **Figure 1A**.

#### Physicochemistry Analysis

The total carbohydrate and uronic acid contents of GP were 82.3% and 10.2%, respectively. After Sevage reagent treatment, the protein content of GP decreased from 23.1 to 7.1%. The sugar components were analyzed by converting the sugars into PMP derivatives. HPLC results showed that GP was composed of galacturonic acid (GalA), glucose (Glc), galactose (Gal), and arabinose (Ara) in a molar ratio of 1.6 to 5.1 to 1.0 to 1.6, respectively (**Figure 1B**).

#### FT-IR Analysis

The GP was analyzed by FT-IR spectroscopy with recorded frequencies ranging from 4,000 to 500 cm^-1^ (**Figure 1C**). A wide absorption peak at 3,446 cm^-1^ in the region of 3,500-3,200 cm^−1^ showed stretching vibrations of O-H and N-H, indicating intramolecular hydrogen bonds. Two peaks in the 3,000-2,800 cm^-1^ region and the 1,400-1,200 cm^-1^ region were angular vibrations of C-H, which indicated that the GP is a polysaccharide compound. The peak at 1,633 cm^-1^ indicates the stretching vibration of the C = O in the acetyl group. The absorption peak at 789 cm^-1^ is the flat pyranose α-type C-H bond in the sugar units.

#### Morphology Analysis

Scanning electron microscopy (100x and 3,000x) showed that the surface of the GP is rough and irregular (**Figure 1D**). It is speculated that this phenomenon may be due to cross-linking and aggregation between molecular chains. It is suggested that there is a strong interaction between the molecules of the samples, and all of them are amorphous. In addition, the GP contained a porous honeycomb structure, which may be formed by the sublimation of ice crystals during freeze-drying or may be related to the side chain of the polysaccharide.

#### Molecular Weight Distribution Analysis

The average weight molecular weight (Mw) of the GP mainly distributed in the range of 10-320 kDa by HPGPC and special GPC software (**Figure 1E**). It was revealed that the polysaccharides have four main peaks with molecular weights of 26, 56, 152, and 275 kDa and distribution percentages of 26.6%, 55%, 13.7%, and 4.8%, respectively. In addition, the average difference in Mw between the peaks was 63 kDa, indicating that the GP is a mixture of a series of hetero-polysaccharides.

### GP Alleviates Intestinal Injury in Rats With DSS-Induced Colitis

**Figure 2:**
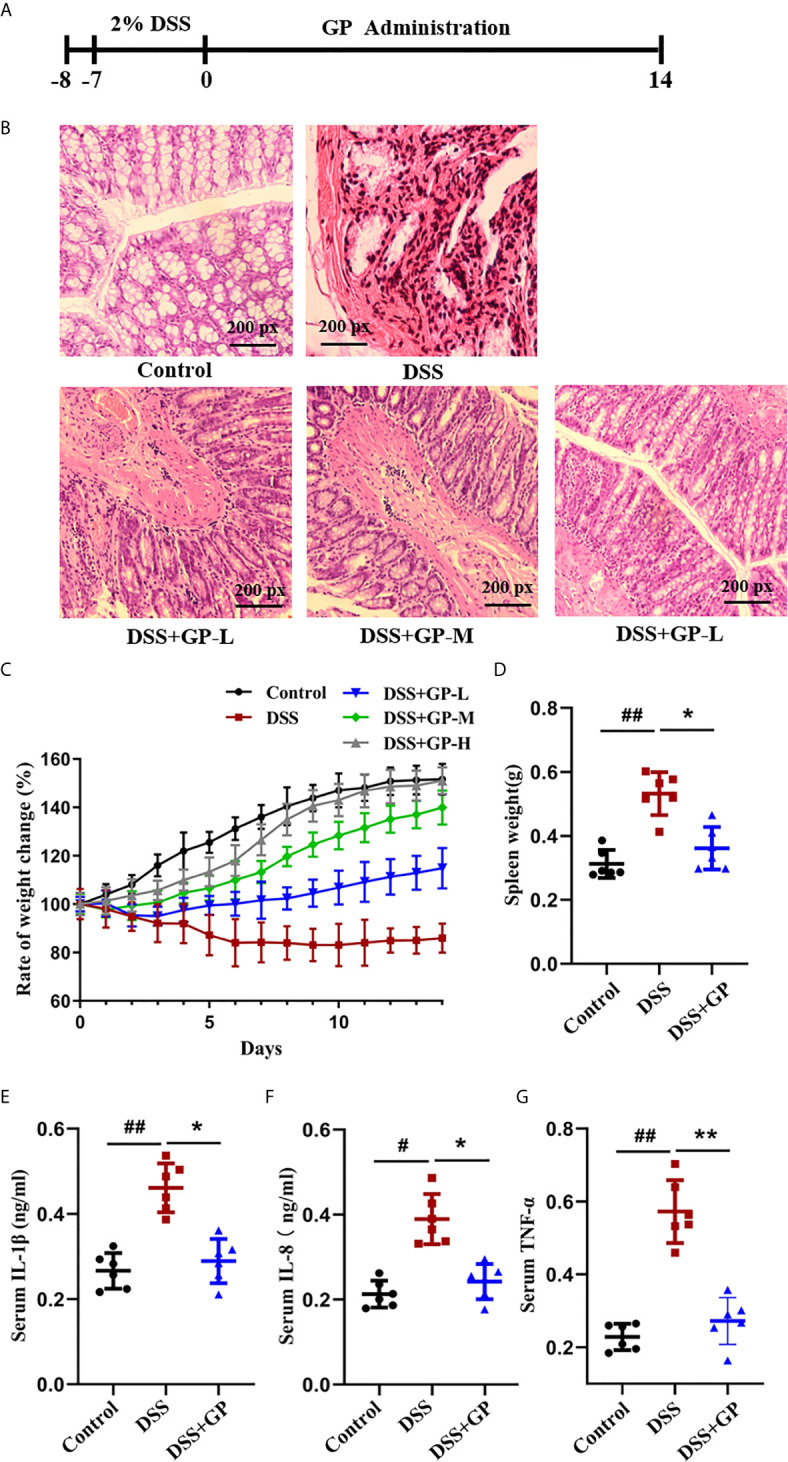
GP alleviated the intestinal inflammation in rats with DSS induced colitis. **(A)** Experimental operation process. **(B)** H&E stained sections of the differently treated rats as described above. **(C, D)** The effects of GP on Rats’ basal body weight and Spleen weight. **(E–G)** Changes of cytokines in different groups *in vivo.* All data shown are representative of 3 independent experiments. Bars in graphs represent mean ± SD, ^#^P < 0.05, ^##^P < 0.01 VS Control group; *P < 0.05, **P < 0.01 VS DSS group.

DSS was used to establish an experimental model of rats with intestinal inflammation as described previously (19, 22). HE stains clearly revealed that GP alleviated severe lesions in colon tissue, such as those that had histopathological characteristics of mucosal damage, necrosis, and inflammatory infiltration in DSS rats (**Figure 2A**). Compared to the control group, the histological observation of the colon of the rats in the DSS model group demonstrated that the inflammatory cell infiltration occurred in the mucosa. The loss of goblet cells and epithelium, distorted crypts, and edema were also found in the DSS model group. After treatment with different doses of GP, the results showed notable histologic improvements in the crypt architecture as well as reductions in edema, mucosal injury, and inflammatory infiltration. In the GP-H group, the histopathological properties of the colon of the rats were the most obviously improved, and ulcer healing lines appeared in some tissues (**Figure 2B**). Furthermore, DSS-treated rats showed profound body weight loss; the change in body weight declined 86.96% when compared to the initial weight. Notably, GP treatment significantly attenuated the loss of body weight. Especially in the high-dose GP group (DSS + GP-H), the change in body weight recovered to 151.05% after 14 days of treatment (**Figure 2C**). Rats in the DSS + GP-L and DSS + GP-M groups also exhibited body weight recovery to certain extents. These results demonstrated that DSS can successfully induce inflammatory intestinal injury in rats, and high-dose GP treatment achieved the best curative effect *in vivo.* Therefore, the high-dose GP group can be used for further studies to obtain more biological information. Additionally, GP treatment also reversed the DSS-induced inflammatory response, as evidenced by the spleen weight and the serum levels of IL-1β, IL-8, and TNF-α (**Figures 2D–G**). (From here forward, the DSS + GP group refers to the DSS + GP-H group if not otherwise mentioned).

### GP Improved the Structure of Gut Microbiota in DSS-Induced Rats

#### Effect of GP on the Diversity of Gut Microbiota

**Figure 3:**
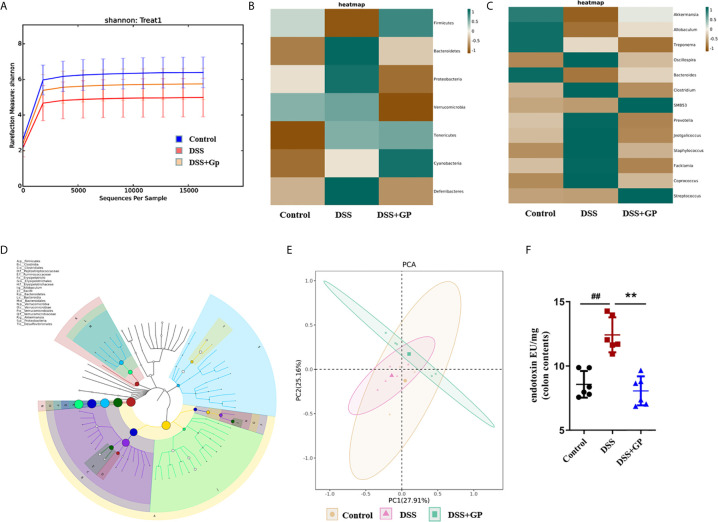
Effects of GP on gut microbiota. **(A)** Rarefaction curves of OTU quantity. **(B)** Heatmap at the level of phylumgenus. **(C)** Heatmap at the level of genus. **(D)** A phylogenetictree of OTUs by GraPhlAn visualization. **(E)** PCA analysis. **(F)** LPS in colon contents. Bars in graphs represent mean ± SD, ^##^P < 0.01 VS Control group; **P < 0.01 VS DSS group.

The Shannon index is a key index used to evaluate microbial community diversity. Compared with the control group, the Shannon index in the model group decreased significantly (*P* < 0.01). The Shannon index of the GP group was significantly higher than that of the model group (*P* < 0.05; **Figure 3A**). The results showed that the microbiota diversity of the model group was lower than that of the normal rats, but GP could restore the microbiota diversity.

#### Effect of GP on Bacterial Community Structure

We analyzed the differences in bacterial abundance in rats before and after GP administration at the phylum and genus levels, and the heatmaps are shown in **Figures 3B, C**. Compared with the blank group, the abundance of Gram-positive Firmicutes bacteria in the model group decreased significantly, while the abundance of Gram-negative *Bacteroidetes, Verrucomicrobia, Proteobacteria, Tenericutes, Cyanobacteria, and Deferribacteres* increased significantly (38). The abundance of Gram-negative bacteria decreased significantly after GP administration. Next, Grapeland was used to construct a hierarchical tree of the composition of the sample population at each classification level to explore the dominant microbial groups (**Figure 3D**). The results showed that the dominant species at the phylum level were *Firmicutes, Bacteroidetes, Verrucomicrobia and Proteobacteria*, and the dominant species at the genus level were *Akkermansia, Allobaculum*, and *Oscillospira*. It is suggested that the above bacteria can be used as a characteristic index for future fecal microbiota transplantation. Community composition analyses at the taxonomic levels of phylum and genus have preliminarily proved that the bacterial community structure before and after GP treatment is significantly different. We further used principal component analysis (PCA) to analyze the overall structure of the flora before and after the occurrence of intestinal inflammation and under the intervention of GP, which also proved that there were significant differences between the three groups (**Figure 3E**).

#### Effect of GP on LPS in Colon Contents

It should be noted that lipopolysaccharide (LPS), one of the components of the cell walls of Gram-negative bacteria in intestinal flora, can cause chronic intestinal inflammation. We determined the endotoxin levels in colon cells (LPS), and they increased 1.45-fold after DSS stimulation. GP treatment reversed the LPS to near the normal level (**Figure 3F**).

Corroborating the above data and the previous reports (32, 39), we demonstrated that the increased levels of LPS were associated with the rising abundance of Gram-negative bacteria in the intestines of the DSS-induced model rats and that GP could regulate the structure of gut microbiota and reduce the abundance of Gram-negative bacteria that produce LPS.

### GP Quenched Intestinal Injury and Restored DSS-Decreased Levels of Autophagy in DSS-Induced Rats

**Figure 4:**
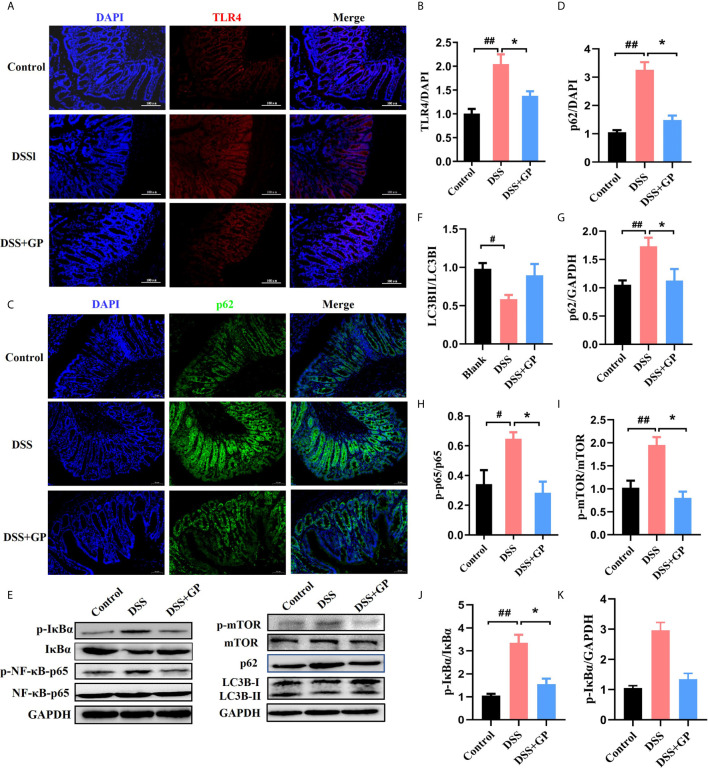
GP inhibits inflammation *via* TLR4 pathway and activates mTOR dependent autophagy in DSS-induced colitis *in vivo*. **(A, C)** Immunofluorescence for TLR4 and p62 was performed on colon sections. **(B, D)** The expressions of TLR4 and p62 were calculated relative to DAPI staining from three independent experiments. **(E)** The expression of autophagy-related and inflammation-related proteins measured by western blot. **(F–K)** The expressions of proteins were quantified by the ratio of phosphorylated protein/total protein and total amount protein/GAPDH. All data shown are representative of 3 independent experiments. Bars in graphs repr Activated TLR4 directly triggers the phosphorylation of mTOR. With the increase of TLR4 in the DSS group, the phosphorylated mTOR was upregulated followed by the dysfunction of autophagy. To investigate whether autophagy was impaired in DSS-induced intestinal inflammation, the expression of autophagy-related proteins (including LC3B, p62, and phosphorylated mTOR) were evaluated by immunofluorescence and Western blot. Compared with the control group, the expression of p62, a cargo protein degraded inside autolysosomes, was upregulated after DSS stimulation, indicating the inhibition of autophagy. Defective autophagy was further confirmed by the decrease in LC3BII/LC3BI when compared with the control group. Autophagy was recovered to a normal level after treatment with GP, indicated by decreased p62 and increased LC3BII/LC3BI levels. These results demonstrated that GP relieved intestinal inflammation by promoting mTOR-dependent autophagy and blocking the inflammatory cascade. Bars in graphs represent mean ± SD, ^#^P < 0.05, ^##^P < 0.01 VS Control group; *P < 0.05 VS DSS group.

When the abundance of Gram-negative bacteria increases, the content of LPS increased consequently and is then recognized by Toll-like receptor 4 (TLR4) on the intestinal cell membrane. This directs a subsequent intracellular reaction and finally leads to an inflammatory response and autophagy inhibition (40). The expression levels of TLR4, IκBα, and NF-κB p65 protein from the rat colon are associated with the inflammatory response and were analyzed by immunofluorescence and Western blot, as shown in **Figure 4**. Compared with the control group, the fluorescence of TLR4 in the model group was significantly enhanced, which indicated that the protein expression level was increased. Notably, GP significantly reversed the DSS-increased expression of TLR4. In addition, the phosphorylation of IκBα was significantly decreased after GP treatment, which suggested that the nuclear translocation of NF-κB was inhibited. As a consequence, GP also reduced the DSS-induced NF-κB activation. In conclusion, GP treatment markedly suppressed DSS-induced intestinal inflammation through the downregulation of TLR4 and the NF-κB pathway.

### Spearman Correlation Analysis of the Bacterial Genera With Highest Abundance and Their Phenotypes

**Figure 5:**
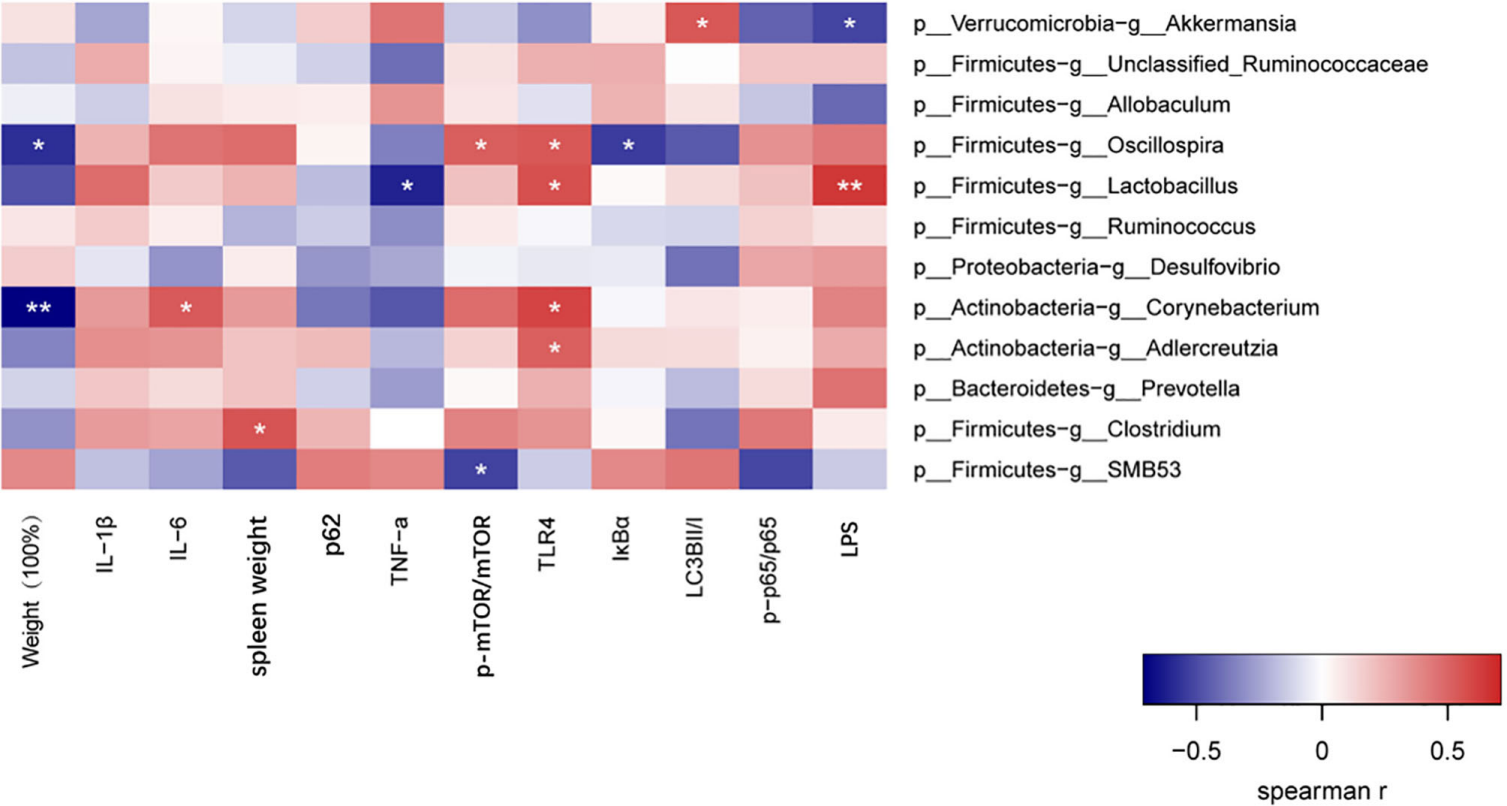
Spearman correlation analysis of the bacterial genera with highest abundance and phenotypes. *P < 0.05, **P < wfi 20.01.

To assess potential association between gut microbiota changes and host autophagy-related phenotypes, we conducted a Spearman correlation analysis of the bacterial genera with the highest abundance and their autophagy-related phenotypes in rats. *p:Akkermansia* from the phylum Verrucomicrobia*−g:Akkermansia* showed significant negative correlation with serum endotoxin, while exhibiting significant positive correlation with LC3BII/LC3BI. On the other hand, there was a highly significant positive correlation between *p_Firmicutes−g_Lactobacillus* and serum endotoxin. *p_Firmicutes−g_Oscillospira, p_*Firmicutes*−g:Lactobacillus, p_Actinobacteria−g_Corynebacterium,and p_Actinobacteria−g_Adlercreutzia* showed significant positive correlation with TLR4 (**Figure 5**). Taken together, these findings indicate that GP treatment inhibited levels of Gram-negative bacteria, inflammation in the intestinal system and enhanced autophagy *via* TLR4.

### Fecal Microbiome Transplantation Supplemented With GP Suppressed Intestinal Inflammation and Improved Autophagy

**Figure 6:**
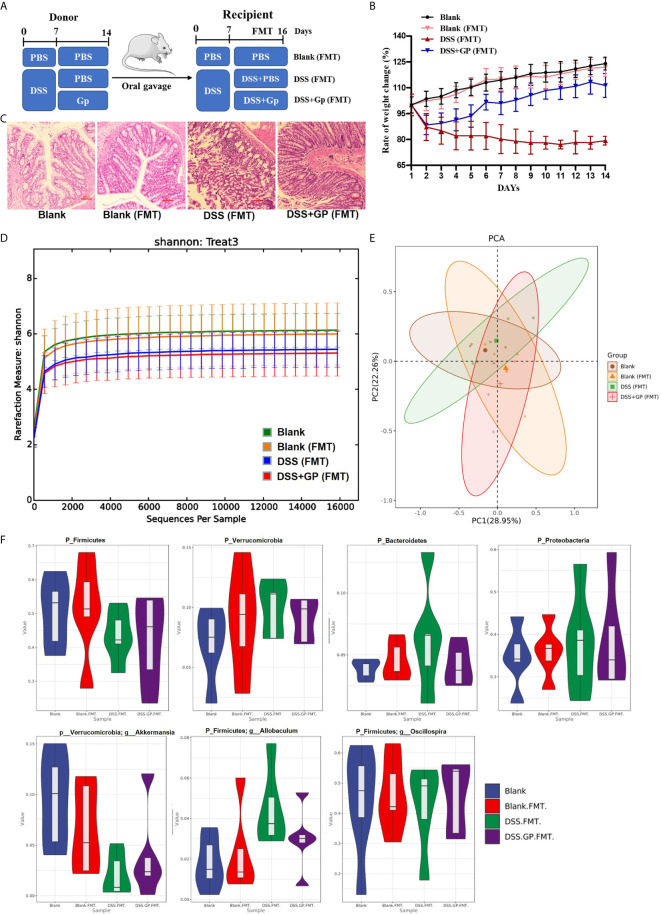
Transplanting of fecal microbiome intervened by GP improves intestinal inflammation. **(A)** The experimental protocol for FMT. **(B)** Changes of body weight (n=10). **(C)** Histopathological changes after HE staining. **(D)** Rarefaction curves of OUT quantity. **(E)** PCA analysis. **(F)** The gut microbiota composition among experimental groups at phylum/genus level.

To validate the potential impact of GP on alleviating intestinal inflammation and on microbe-autophagy interactions, we performed FMT experiments by using oral gavage in rats (**Figure 6A**). 20 rats were randomly divided into four groups: DSS (FMT), DSS + GP (FMT), blank (FMT), and blank (normal rats). The body weight changes of rats in the DSS (FMT), DSS + GP (FMT), blank (FMT), and blank groups are shown in **Figure 6B**. Blank (FMT) and blank groups showed similar body weight changes, which indicated that the FMT operation had little effect on the mice. The body weight of the rats transplanted with DSS-treated microbiota decreased significantly, while the rats transplanted with DSS+GP (FMT)-treated microbiota recovery the body weight. HE staining of pathological sections showed that the colon epitheliums of the blank (FMT) and blank group were intact without hyperemia, edema, hemorrhage, or inflammatory cell infiltration. In the DSS (FMT) group, the intestinal epithelial structure was incomplete, and crypt abscesses, a reduction in goblet cells, and large number of neutrophil infiltrations were observed. Compared with the DSS (FMT) group, the colon epithelial cells in the DSS + GP (FMT) group were less damaged, and the crypt abscesses and inflammatory infiltrations were relieved (**Figure 6C**).

The bacterial profiles of the donor mice were measured using 16S rRNA sequencing to investigate the effects of GP on the fecal microbiome of mice. The Simpson Diversity Index of the operational taxonomic unit level in the DSS + GP (FMT) group was significantly higher than that of the DSS (FMT) group, indicating that GP alters the richness and diversity of the microbial community. Significant separations were observed among the four groups during PCA (**Figure 6D**), which is similar to the result of hierarchical clustering. Further PCA showed that the overall structure of the microbiota had significant differences in the four groups (**Figure 6E**). The comparison of gut microflora with high abundance at the phylum and genus levels in each group is shown in **Figure 6F**. Compared with blank group, there was no significant change in the phylum and genus levels of blank (FMT) bacteria. Compared with blank and blank (FMT) groups, the abundance of *p_ Firmicutes* and *p_Verrucomicrobia-g_Akkermansia* in the DSS (FMT) group was significantly lower, while the abundance of *p_Verrucomicrobia*, *p_Bacteroidetes, p_Proteobacteria*, and p_Firmicutes-g_*Allobaculum* were significantly higher. The abundances of the above bacteria in the DSS + GP (FMT) group and the DSS (FMT) group showed the opposite trend of change except *p_Firmicutes-*g *_Oscillospira*.

**Figure 7:**
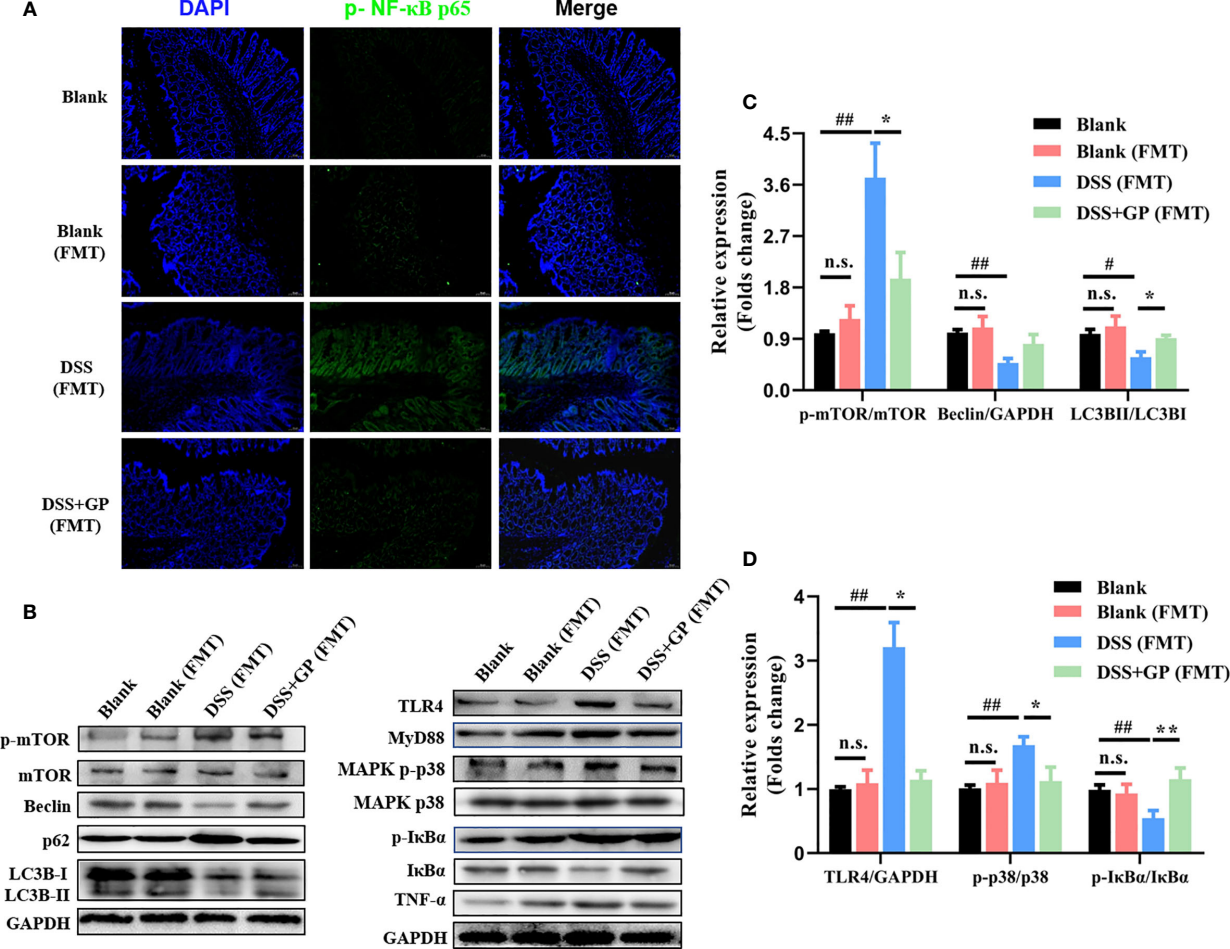
Transplanting of fecal microbiome intervened by GP improves intestinal inflammation. **(A)** Immunofluorescence for p-NF-κB was performed on colon sections. **(B)** The expression of autophagy-related and inflammation-related proteins measured by western blot. **(C, D)** The expressions of proteins were quantified by the ratio of phosphorylated protein/total protein and total amount protein/GAPDH. All data shown are representative of 3 independent experiments. Bars in graphs represent mean ± SD, ^#^P < 0.05, ^##^P < 0.01 VS Control group; *P < 0.05, **P < 0.01 VS DSS group, n.s., no significant differences.

To validate the protective effect of GP on intestinal inflammation associated with the microbe-TLR4-autophagy axis, we detected the levels of several key proteins using Western blot analysis. FMT had almost no effects on the TLR4 pathway and mTOR-dependent autophagy, as shown in **Figure 7**. Meanwhile, the rats transplanted with DSS-treated microbiota showed a marked activation of the classic TLR4-p38 MAPK-NF-κB inflammatory pathway (**Figures 7A, B**) as well as the phosphorylation of mTOR. Subsequently, the accumulation of p62 and the decrease of LCBII/LC3BI suggested defective autophagy. Notably, there was significant attenuation of TLR4 and degradation of p62 after FMT with GP-treated microbiota, which achieved similar therapeutic effects compared to GP lavage. In summary, the potential mechanism of GP on ameliorating intestinal inflammation *via* regulation of the gut microbiota and which was associated with TLR4-autophagy.

### Blocking TLR4-mTOR Ameliorates Inflammation *via* Promoting Autophagy in LPS-Induced HT-29 Cell

**Figure 8:**
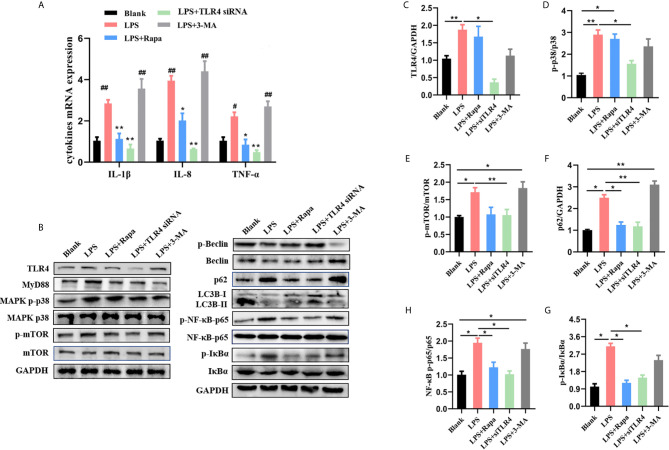
Blocking upstream TLR4-MAPK pathway activated autophagy and quenched inflammation. **(A)** Changes of cytokines contents in different groups in LPS-induced HT-29 cells. **(B)** The changes of autophagy-related and inflammation-related proteins measured by western blot. **(C–H)** The expressions of proteins were quantified by the ratio of phosphorylated protein/total protein and total amount protein/GAPDH. All data shown are representative of 3 independent experiments. Bars in graphs represent mean ± SD, *P < 0.05, **P < 0.01. ^#^P < 0.05, ^##^P < 0.01 VS Control group.

The relationship between intestinal flora disturbance and TLR4-autophagy axis has been widely accepted (41, 42), and gut-derived lipopolysaccharides (LPSs) seem to be the linkage between them. According to the *in vivo* results, related mechanisms of gut microbiota-TLR4-autophagy regulation were subsequently explored in LPS-induced HT-29 cells. To explore whether the release of NF-κB-induced cytokines and oxidative stress were associated with autophagy, we chose 3-MA and rapamycin as the inhibitor and activator of autophagy, respectively. Firstly, pro-inflammatory cytokines were determined as shown in **Figure 8A**. When cells were treated with one μg/mL LPS for six hours, IL-8 and IL-1β levels increased by nearly three-fold; these levels ameliorated to a certain extent after rapamycin treatment (an autophagy activator). When autophagy was inhibited by 3-MA, the release of LPS-induced inflammatory cytokines dramatically increased even higher than that of the LPS-induced group. Secondly, the expressions of p62, LC3B, and Beclin-1 were determined by Western blot analysis (**Figure 8B**). In the second lane of **Figure 8**, it can be seen that LPS blocked autophagy *via* the activation of mTOR and activated an inflammatory transcription factor, which is consistent with previous reports (19, 22). While the expression of Beclin-1, degradation of p62, and conversion of LC3B were increased significantly in the rapamycin groups, this was also accompanied with the inhibition of NF-κB p65. As expected, suppression of autophagy by 3-MA significantly inhibited the level of autophagy and dramatically deteriorated the inflammation. The results above indicated that impaired autophagy was involved in LPS-induced inflammation in HT-29 cells.

Recent research has focused on Toll-like receptors (TLRs), which might be involved in orchestrating LPS-induced autophagy inhibition and inflammation aggravation *via* mTOR (19). Thus, we next sought to investigate whether TLR4 is a potential target of governance of the activation of autophagy. As shown in **Figure 8**, LPS significantly upregulated the expression of TLR4 and its downstream signaling molecules MyD88 and p38 MAPK in HT-29 cells, which coincided with high phosphorylation levels of mTOR and inhibition of autophagy. Autophagy-related proteins in TLR4 siRNA transfected cells further confirmed the regulation role of TLR4. TLR4 siRNA attenuated the autophagy impairment and inflammation activation induced by LPS, while 3-MA and rapamycin exhibited almost no effect on TLR4-MyD88. Corroborating the data above, we demonstrated that LPS-decreased autophagy was governed by the upstream TLR4-mTOR pathway. Additionally, autophagy was involved in inhibiting cytokine secretion and protecting cells from oxidative stress.

## Discussion

Inflammatory bowel disease (IBD) is a kind of chronic, nonspecific intestinal inflammation, which includes ulcerative colitis and Crohn’s disease. Acetaminophen and non-steroidal anti-inflammatory drugs are the primary clinical treatment, but long-term usage of these can cause kidney and digestive system damage. It is urgent to develop effective treatment strategies, including therapeutics and functional foods.

P. ginseng is a perennial herb. It has been used for thousands of years in China, Japan, and Korea as a traditional medicine. Its chemical properties and pharmacological activities have drawn attention from across the world. Ginseng is available in liquid or solid forms for consumption, and its anti-inflammatory effects have been reported in many studies. Modern pharmacological studies have shown that P. ginseng has therapeutic effects on intestinal system diseases, such as ulcerative colitis, Crohn’s bowel disease, and intestinal cancer (43). The polysaccharide is an important active component of P. ginseng in mediating the inflammatory response and immune function and has obvious inhibitory effects on many pathogenic bacteria (44). In this study, polysaccharide from P. ginseng was isolated and simi-purified, and its basic physicochemical properties were studied by HPLC, FT-IR, and GPC. The morphology of the polysaccharide was also characterized by SEM analysis. Further, the activity of GP on intestinal inflammation was evaluated by a model of DSS-induced intestinal inflammation in rats. Our results showed that the weight loss of rats in the GP treatment group was inhibited, and the intestinal mucosa loss as well as the infiltration of inflammatory cells in the lamina propria was reduced in the treatment group after the administration of GP. This indicates that GP has a therapeutic effect on DSS-induced colitis in rats, but the specific mechanism is still unclear.

There are at least 10^14^ microorganisms that reside in human intestines which are involved in the host’s immune response, metabolism, and homeostasis maintenance (45). A large number of clinical and experimental studies have shown that many natural plant polysaccharides can play a therapeutic role in intestinal inflammation by regulating the imbalance of gut microbiota (46). Similar results were found in this study: compared with the normal group, the model group of rats had obvious intestinal flora imbalances, while the administration of GP caused a recovery effect on the gut microbiota in model rats. Compared with the model group, the abundance of Gram-negative bacteria decreased significantly after GP administration. It is clear that inflammatory bowel disease is closely related to the regulation of the structure and composition of the intestinal microbiota; however, the pathways involved in the regulation and how to remedy the change in intestinal flora still required elucidation. At present, although there are reports that polysaccharides from P. ginseng can regulate intestinal flora and play a therapeutic role in inflammatory bowel disorders such as IBD and UC, this conclusion overemphasizes the role of intestinal flora and fails to describe the causal mechanism of intestinal flora in the process of disease treatment.

**Figure 9:**
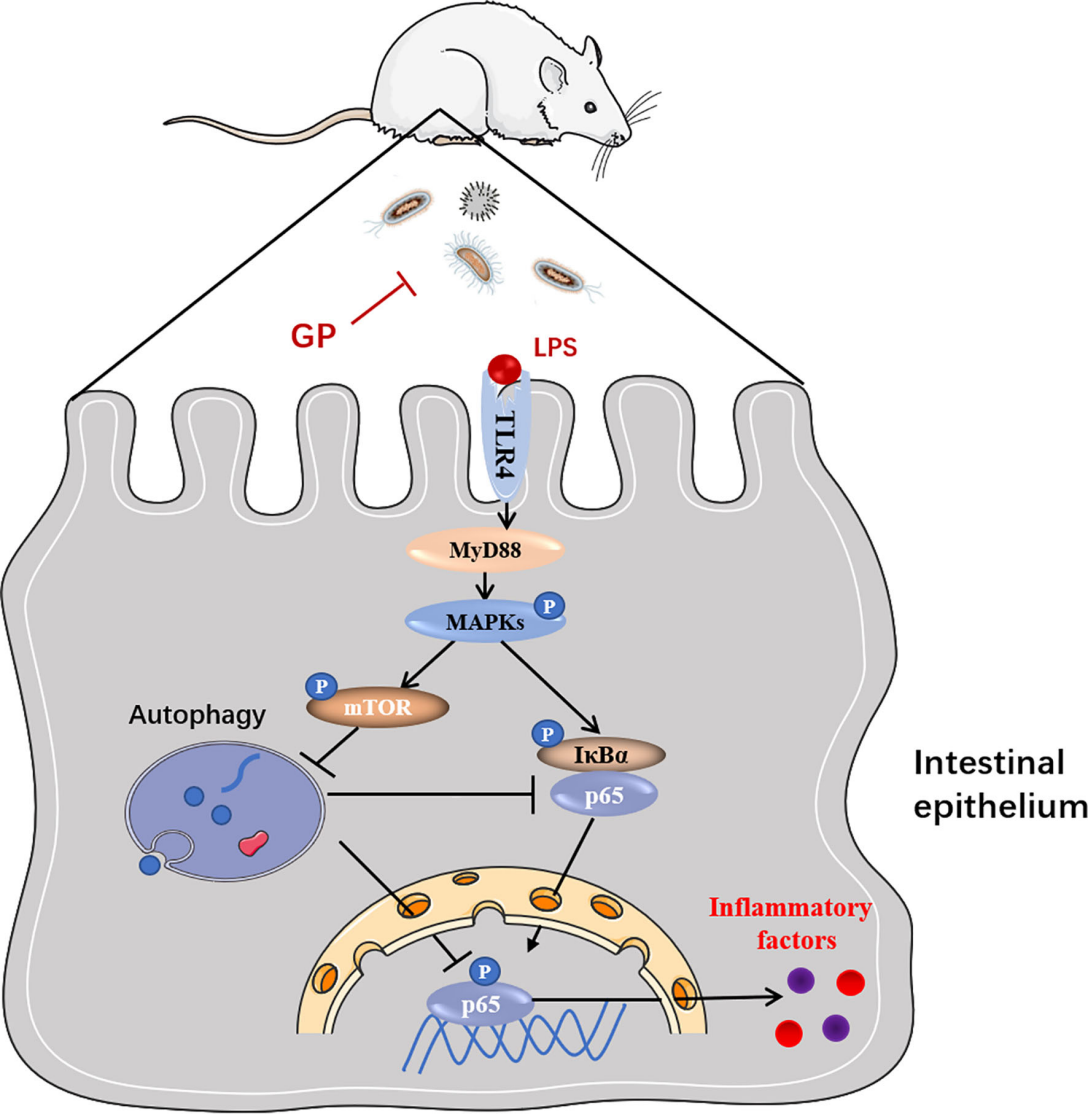
Schematic representation of the proposed mechanism of GP on ameliorating intestinal inflammation.

Gut microbiome disorder is closely related to the incidence of a variety of intestinal diseases. In the pathological state of inflammatory bowel disease, the chronic accumulation of activated neutrophils, macrophages, and dendritic cells leads to a change in intestinal flora structure, especially the proliferation of many Gram-negative opportunistic pathogens. Lipopolysaccharide (LPS) is an essential component of the cell wall of Gram-negative bacteria that is involved in triggering the inflammatory cascade (2). Gut microbiota-derived Lipopolysaccharides is closely related to the intestinal flora and abnormally activates the host immune response through TLR4. TLR4 is the receptor for LPS and is of great importance to the self-repair of intestinal epithelial cells (47). Activated TLR4-induced phosphorylation of IκBα *via* the downstream signaling molecule MyD88 eventually leads to the translocation of NF-κB and the release of cytokines including IL-lβ, IL-8 and TNF-α. TNF-α is an important initiator of inflammation, which can cause a microcirculatory disturbance of colonic mucosa and weaken the mucosal barrier, leading to mucosal damage and inflammatory cell infiltration (48). Clinical studies also confirm that the level of TNF-α in serum is positively correlated with the severity of IBD (49). IL-1β increases the cytokines produced by macrophages, such as IL-6, TNF-α, and IL-8, which can promote the aggregation of neutrophils to inflammatory sites and then cause intestinal mucosal tissue damage as well as an intestinal inflammatory response (50). Thus, blocking the TLR4 pathway is an alternative strategy for the development of anti-intestinal inflammation drugs or functional foods (51). In our study, GP markedly suppressed DSS-increased expression of TLR4 and reduced the recruitment of downstream proteins, which was accompanied by decreased cytokines and also the repairment of intestinal mucosa. Our data confirmed that the therapeutic effect of GP on intestinal inflammation is associated with the downregulation of TLR4 expression (**Figure 9**).

Autophagy, a highly conserved process that evolved in eukaryotes, is involved in maintaining organism homeostasis *via* lysosome-mediated self-digestion and recycling of organelles and proteins. Although the pathogenesis of IBD is ambiguous, studies have pointed out that autophagic dysfunction is a key factor in the persistence of intestinal inflammation (19). Pioneer evidence from genome-wide association studies suggested that defective autophagy is associated with an increased risk of IBD (52, 53). Several roles of autophagy in gut inflammation have been summarized: elimination of pathogens, regulation of antigen presentation, governance of the secretion of cytokines, and maintenance of lymphocyte homeostasis. Notably, the accumulation of p62 in DSS-induced rats confirms the dysfunction of autophagy in the pathologicalprocess of intestinal inflammation. After treatment with GP, both groups of rats with intestinal inflammation presented diminished levels of phosphorylated mTOR, LC3B, and Beclin-1, which paralleled with a decrease of p62.

On the basis above, we further discussed the interaction between gut microbiome, TLR4 and autophagy, aiming to reveal the potential mechanism of the impact of GP on intestinal inflammation. In this study, 16S rRNA sequencing was used to evaluate the changes in intestinal flora before and after GP administration. The Spearman correlation coefficient was used to evaluate the correlation between high abundances of bacteria at the genus level and important characteristics of intestinal inflammation in order to explore the correlation between intestinal flora and autophagy, inflammation and oxidative stress. The results showed that the expression of TLR4 was positively correlated with four of the bacteria with high abundance in intestinal flora*. p:*Verrucomicrobia*−g:Akkermansia* showed significant negative correlation with serum endotoxin, while displaying significant positive correlation with LC3BII/I. It can be seen that changes in intestinal flora, TLR4 pathway activation, and autophagy disorder are connected together in the occurrence and development of intestinal inflammation.

In recent years, the focus of research on intestinal flora has changed from association to modulation (54). The most convincing experimental evidence of the role of intestinal microbiota in human diseases can be obtained from relevant experiments of intestinal flora transplantation (FMT) (55). However, there are few studies on the treatment of inflammatory bowel disease by FMT with the addition of GP. Therefore, indirect experimental animal models are needed to establish causal relationships between altered microbiomes and disease pathogenesis. In this study, FMT-related experiments showed that GP could improve the symptoms of DSS-induced intestinal inflammation by inhibiting the TLR4-NF-κB pathway and activating mTOR-dependent autophagy.

Recently, emerging research has focused on the connection between inflammation and autophagy (21). Accumulating evidence indicates that inflammation and autophagy are linked by reciprocal regulation, and they are orchestrated by the upstream TLR4-mTOR pathway. It has been reported that inhibiting autophagy is a novel role of the TLR4-MyD88 in intestinal epithelial dysfunction. Subsequently, the release of NF-κB-induced cytokines was orchestrated by autophagy. In addition to previous research, our data also demonstrated that TLR4-MyD88 is involved in the regulation of mTOR phosphorylation, and that mTOR is a newfound intersection between inflammation and autophagy. Blocking the TLR4 pathway not only promotes the repair of autophagy, but also suppresses the disaggregation of IκBα-NF-κB complex through inhibiting the phosphorylation of IκBα. The transcription factor NF-κB is present an inactive state complex with the inhibitory IκB proteins (56). Phosphorylation of IκBα results in the release and nuclear translocation of active NF-κB (57). Consequently, diverse group of extracellular signals are activated including inflammatory cytokines, growth factors, and chemokines. Importantly, autophagy also participates in reducing the degradation of the IκBα-NF-κB complex (58). Treatment with 3-MA deteriorated the LPS-induced inflammatory response in HT-29 cells. Meanwhile, rapamycin, an autophagy activator, ameliorated inflammation to a great extent, which further indicates that autophagy is involved in suppressing NF-κB activation.

Therefore, for the first time, this study elucidated that polysaccharide purified from P. ginseng ameliorates intestinal inflammation through regulating gut microbiota by experiments with FMT and repaired the defective autophagy. Our research revealed the mechanism concerning the intestinal anti-inflammatory effect of GP and provided a promising therapy for IBD.

## Conclusion

The present results highlighted that the purified polysaccharides from P. ginseng showed intestinal anti-inflammatory effects in DSS-induced rats by regulating gut microbiota and mTOR-dependent autophagy. It was suggested that GP-treated intestinal flora transplantation elevated autophagy and suppressed inflammatory response that was associated with the downregulation of LPS-producing bacteria. Consequently, NF-κB-induced inflammation was attenuated *via* the activation of mTOR-dependent autophagy. The results suggested the view that LPS maybe the potential linker between microbiota and autophagy, and GP may have potential in intestinal inflammation disease therapies, providing a foundation for the potential utilization of polysaccharide from P. ginseng for functional foods and complementary medicines.

## Data Availability Statement

The datasets presented in this study can be found in online repositories. The names of the repository/repositories and accession number(s) can be found in the article/supplementary material.

## Ethics Statement

The animal experiment protocol (No. 20200128006) was approved by the Changchun University of Chinese Medicine. Written informed consent was obtained from the owners for the participation of their animals in this study.

## Author Contributions

DW is the first authors. MW obtained funding. MW, DW, and SS designed the study. YZ and DZ collected the data. MW and DW drafted the manuscript. All authors contributed to the article and approved the submitted version.

## Funding

This work was supported by the National Key Research and Development Program of China [2017YFC1702100], the National Natural Science Foundation of China [81603276, U19A2013 and 82004099], the Department of Science and Technology of Jilin Province [20190101010JH, 20200201419JC and 202002053JC], and the Science and Technology Projects in Jilin Province Department of Education [JJKH20200903KJ].

## Conflict of Interest

The authors declare that the research was conducted in the absence of any commercial or financial relationships that could be construed as a potential conflict of interest.
